# Clinicopathological and molecular characteristics associated with pathological complete response in neoadjuvant immunotherapy for breast cancer

**DOI:** 10.3389/fimmu.2026.1771228

**Published:** 2026-03-27

**Authors:** Buchen Zhang, Zhuo Chen, Runzhi Mao, Jinfeng Zhu, Wenjie Cai, Bin Zhao

**Affiliations:** 1 Quanzhou First Hospital Affiliated to Fujian Medical University, Quanzhou, China; 2 Ruijin Hospital, Shanghai Jiao Tong University School of Medicine, Shanghai, China; 3 The Second People’s Hospital Affiliated With Fujian University of Traditional Chinese Medicine, Fuzhou, China

**Keywords:** biomarker, breast cancer, immune checkpoint blockade, neoadjuvant therapy, pathologic complete response

## Abstract

**Background:**

In breast cancer, immune checkpoint inhibitor (ICI)-based neoadjuvant therapy has been approved for clinical practice since 2021. Nonetheless, the predictive values of routinely collected clinicopathological and molecular characteristics in neoadjuvant immunotherapy remain unknown.

**Methods:**

We searched EMBASE and MEDLINE databases for randomized controlled trials (RCTs) comparing ICI-based neoadjuvant therapy with conventional treatment. The primary outcome was pathological complete response (pCR). The odds ratio (OR) and its 95% confidence intervals (CIs) were calculated. This meta-analysis was registered in PROSPERO (CRD420261307112).

**Results:**

Here, with 5674 patients enrolled in 12 RCTs, our study revealed ICI-based neoadjuvant therapy was associated with significantly increased pCRs (OR, 1.59; 95% CI, 1.32-1.90; *P* < 0.001). Notably, neoadjuvant immunotherapy did not demonstrate a statistically significant improvement of pCRs in patients with HER2+ tumors (OR, 1.17; 95% CI, 0.92-1.49; *P* = 0.23). Moreover, the interactions between ICI-based neoadjuvant therapy and nodal status (*P_Interaction_* < 0.001) or stromal tumor-infiltrating lymphocyte (sTIL, *P_Interaction_* = 0.05) were statistically meaningful. More pCRs were observed in patients with nodal-positive (OR, 1.89; 95% CI, 1.56-2.28; *P* < 0.001) or high-density sTIL tumors (OR, 2.89; 95% CI, 1.49-5.61; *P* < 0.001), but not in women with nodal-negative (OR, 1.05; 95% CI, 0.80-1.39; *P* = 0.71) or low-density sTIL tumors (OR, 1.26; 95% CI, 0.73-2.17; *P* = 0.41). There was insufficient evidence to support other characteristics, including race, age, PD-L1 expression, clinical stage, hormone receptor (HR) status, menopausal status, Eastern Cooperative Oncology Group (ECOG) performance status, and T stage, as predictive biomarkers to guide patient selection for neoadjuvant immunotherapy in breast cancer.

**Conclusion:**

Neoadjuvant immunotherapy was associated with favorable outcomes in breast cancer. However, for patients with HER2+, nodal-negative, or low-density sTIL tumors, clinicians need to carefully balance efficacy, safety, and patient preferences to deliver individualized treatment. Further randomized trials with long-term outcomes are needed to confirm the predictive values of these biomarkers.

**Systematic Review Registration:**

https://www.crd.york.ac.uk/PROSPERO/view/CRD420261307112, identifier PROSPERO CRD420261307112.

## Introduction

Globally, breast cancer (BC) is the most common malignancy diagnosis and the second leading cause of female mortality (1). Currently, due to the development of systematic therapies and advancements in surgery, the standard approach to BC treatment has progressively transitioned to neoadjuvant therapy. In the past decade, the development of immune checkpoint inhibitors (ICIs) targeting PD-1/PD-L1 has revolutionized cancer treatment (1). Recently, emerging evidences suggest that applying ICIs in the pre-surgical phase, with the tumor still present, might be ideal (2). In fact, the US Food and Drug Administration (FDA) granted the clinical application of neoadjuvant chemo-immunotherapy for patients with triple-negative breast cancer (TNBC) in 2021 due to the success of KEYNOTE-522 (3).

Identifying the optimal pre-surgical management approach for BC is a comprehensive process (1). The strategy should focus on addressing tumor heterogeneity and varied responses to neoadjuvant therapies, enhancing pathological complete response (pCR) rates, lowering the risk of recurrence in high-risk early BC, and establishing response-adjusted treatment strategies that promote personalized care and de-escalation of therapy in certain specific populations (2). Therefore, research efforts have concentrated on finding potential biomarkers to enable accurate patient selection and prognosis, thus preventing non-responders from experiencing treatment-related adverse events. Indeed, in lung cancer, the European Medicines Agency (EMA) has approved nivolumab-based immunotherapy in the neoadjuvant phase exclusively for patients with tumor cell PD-L1 expression levels over 1% (4). Despite being a biologically plausible biomarker for predicting tumor response and survival prognosis, the role of PD-L1 expression in ICI-based neoadjuvant therapy has not been systematically explored in BC.

Given the significant heterogeneity in BC, it is crucial to fully comprehend the clinical implications and address the controversial aspects of neoadjuvant immunotherapy before solely depending on this management approach. This study aims to explore how routinely gathered data on patients, disease, and molecular features can predict which BC patients may benefit from neoadjuvant immunotherapy. However, due to the design and power limitations, individual randomized clinical trials (RCTs) cannot effectively show treatment differences between patient subgroups based on clinical-pathological or molecular characteristics. Accordingly, we conduct a meta-analysis of RCTs comparing ICI-based neoadjuvant therapy with conventional treatment, with pCR as the primary endpoint, to provide critical insights into this clinically important need.

## Method

This study was reported according to the PRISMA guideline (5) and was registered in PROSPERO (CRD420261307112).

### Search strategy and selection criteria

A systematic search of EMBASE and MEDLINE databases for published trials on neoadjuvant immunotherapy, alone or in combination, in breast cancer from inception to November 2025 was carried out with no language restriction. The keywords included: adebrelimab, atezolizumab, avelumab, camrelizumab, cemiplimab, dostarlimab, durvalumab, envafolimab, ipilimumab, nivolumab, pembrolizumab, relatlimab, sintilimab, sugemalimab, tremelimumab, toripalimab, tislelizumab, breast cancer, clinical trial, and neoadjuvant therapy. All investigators independently conducted the initial search, reviewed the title and abstract for relevance, and classified the potential studies as included, excluded, or uncertain. For uncertain studies, the full texts were checked to verify eligibility.

Standards for inclusion and exclusion were established beforehand. Studies were required to fulfill the following criteria to be considered eligible: (1) study design: RCTs irrespective of clinical phase; (2) population: individuals aged over 18 years old with histologically confirmed resectable breast cancer; (3) intervention: ICI-based neoadjuvant therapy was administered in at least one arm of patients regardless of the dosage or treatment duration; and (4) outcomes: available information on pCR, defined as ypT0/is ypN0, in all enrolled population and/or subgroups classified by various clinicopathological or molecular characteristics. Excluded studies were those that were: (1) other research on this subject, including retrospective studies, review articles, pre-clinical papers, editorials, comments, quality of life studies, phase I and non-randomized phase II trials, and cost-effectiveness analyses; (2) research concerning pediatric patients or individuals with blood disorders; and (3) individuals with active brain metastases, autoimmune conditions, and those taking glucocorticoids or immunosuppressive drugs.

### Data extraction and quality assessment

A prespecified form was applied by all investigators to independently extract the following information: (1) study information, including trial name, study design, clinical phase, neoadjuvant treatment regimens, primary endpoints, and the intention-to-treat sample size; (2) baseline characteristics of the included patients, including age, race, clinical stage, cancer subtype, tumor node metastasis classification (TNM) stage, nodal status, hormone receptor status, menopausal status, Eastern Cooperative Oncology Group (ECOG) performance status, and PD-L1 expression status; (3) outcomes, including the number of patients who achieved pCR overall or subgroups classified by various clinicopathological or molecular characteristics. Odds ratios (ORs) and their 95% CIs were calculated or extracted from each included study. For trials with multiple publications, we only included the latest or most comprehensive report. The assessment of bias risk was conducted with the Cochrane risk of bias tool (6).

Whenever disagreements arose concerning study selection, data extraction, and risk of bias assessment, all investigators held discussions to address the issues. The discrepancies were settled once all authors reached a consensus.

### Statistical analysis

The main objectives here were to evaluate the improvements of pCR rates among BC patients with various clinicopathological and molecular features undergoing neoadjuvant immunotherapy. The *I*^2^ statistic was applied to evaluate the extent of inconsistency contributing to the heterogeneity across different trials. The assumption of homogeneity was deemed invalid for *I*^2^ > =50% and *P* < 0.10. To estimate the magnitude of the treatment benefit, we pooled results with either the fixed-effects inverse-variance-weighted method or random-effects models based on the level of heterogeneity. Tests of interaction were conducted to determine the differences in treatment and expressed as *P* for interaction (7). Pre-defined subgroup analyses were conducted to investigate potential sources of heterogeneity and assess how different exclusion criteria affect the overall effectiveness of neoadjuvant immunotherapy. In this study, the sensitivity analysis was conducted according to different cancer types, masking methods, sample size, drug target, clinical stage, and clinical phase.

To assess publication bias, Begg’s funnel plots were visually inspected (8). Additionally, the Egger linear regression test and the Begg rank correlation test were performed, with significance set at *P* < 0.10 (8, 9).

All analysis was conducted by Stata 19.0 and MedCalc 18.2.1. Two-sided P<0.05 was considered statistically significant. All 95% CIs were two-sided.

## Results

### Baseline characteristics

**Figure 1:**
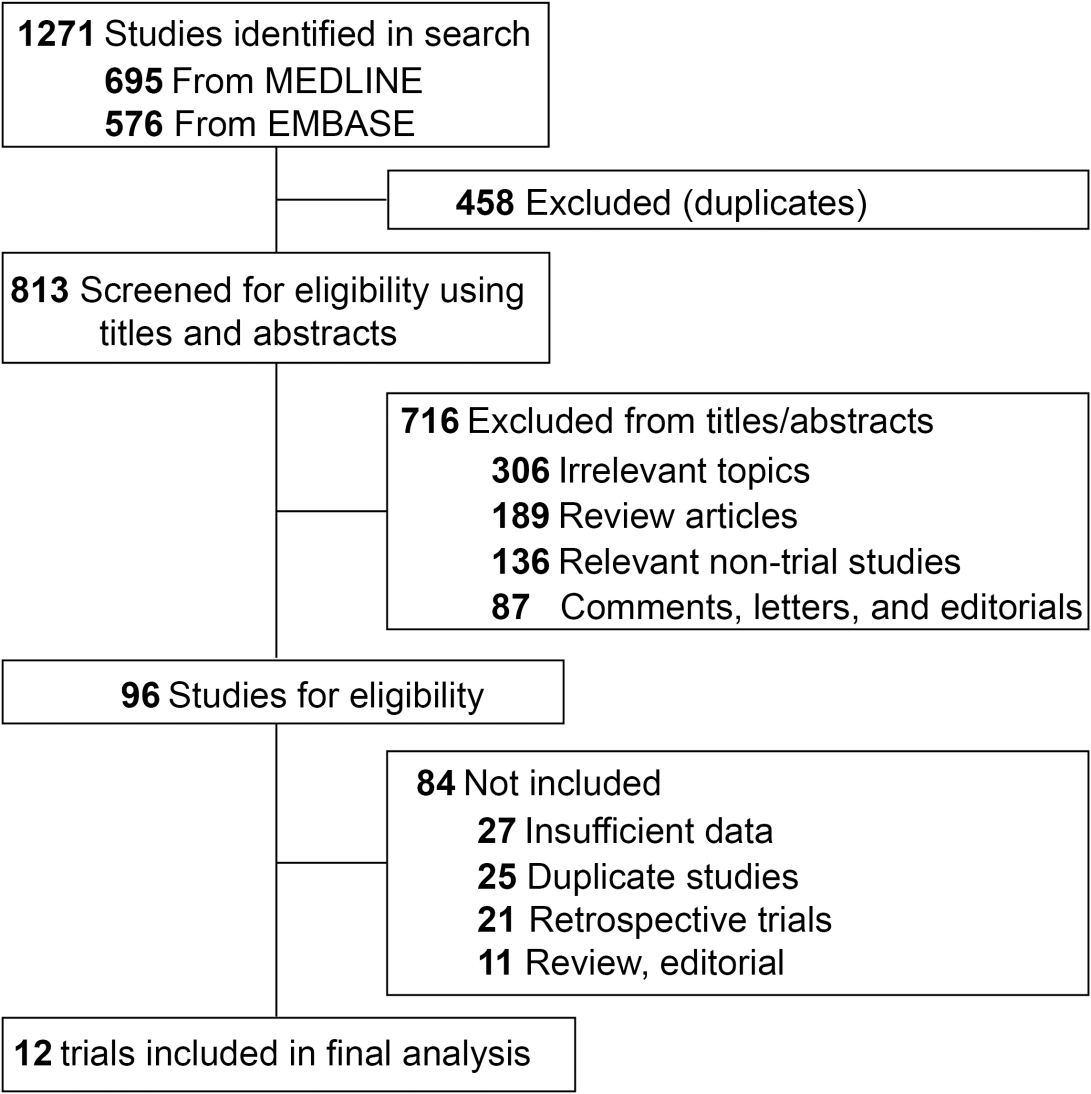
Flow-chart diagram of selected clinical trials included in this study.

1271 potentially relevant articles were identified from the initial search. 458 papers were excluded due to duplication in both MEDLINE and EMBASE databases. 716 studies failed to meet the inclusion criteria after screening the titles and abstracts. Further review of the full-text removed 84 articles (**Figure 1**).

Totally, 12 RCTs with 5674 patients met the inclusion criteria (**Table 1**), namely ABCSG-52 (10), APTneo (11), CamRelief (12), CheckMate 7FL (13), GeparNuevo (14), IMpassion031 (15), IMpassion050 (16), KEYNOTE-522 (3), KEYNOTE-756 (17), I-SPY2 (18), NCI 10013 (19), and NeoTRIP (20). The number of enrolled patients ranged from 58 in NCI 10013 (19) to 1278 in KEYNOTE-756 (17). Four trials, including GeparNuevo (14), I-SPY2 (18), NCI 10013 (19), and ABCSG-52 (10), were phase II RCTs; the other eight trials were phase III studies. Patients with early BC were recruited in all eligible trials, while locally advanced BC patients were only enrolled in APTneo (11), CamRelief (12), and NeoTRIP (20). The primary endpoint was pCR in all RCTs except NeoTRIP (20) and APTneo (11); EFS was a co-primary endpoint in KEYNOTE-522 (3) and KEYNOTE-756 (17). The median follow-up ranged from 6.6 months in NCI 10013 (19) to 54.0 months in NeoTRIP (20). These RCTs were conducted in six studies with 2463 TNBC patients, three studies with 2038 HR+/HER2- women, and three studies with 1173 HER2+ subjects. Across all 5674 patients (median age range, 48–58 years), 2576 patients (45.4%) were in the control arms, 3098 patients (54.6%) were treated with ICIs. Among them, 1488 women were treated with pembrolizumab, 1043 with atezolizumab, 257 with nivolumab, 222 with Camrelizumab, and 88 with durvalumab.

**Table 1:** Baseline characteristics of eligible randomized trials.

Study	Masking	Phase	Cancer subtype	Clinical stage	Key inclusion criteria	Primary endpoint	Treatment	No. of patients
GeparNuevo (14)	Double-blind	II	TNBC	Early BC	cT1b-4cN0-3	pCR	Durvalumab + carboplatin + paclitaxel	88
							Carboplatin + paclitaxel	86
IMpassion031 (15)	Double-blind	III	TNBC	Early BC	cT2-4cN0-3	pCR	Atezolizumab + nab-paclitaxel	165
							Nab-paclitaxel	168
IMpassion050 (16)	Double-blind	III	HER2+	Early BC	cT2–4 cN1-3	pCR	Atezolizumab + doxorubicin/Cyclophosphamide	228
							Doxorubicin/Cyclophosphamide	226
I-SPY2 (18)	Open-label	II	HER2-	Early BC	cT2–4 cN0-3	pCR	Pembrolizumab + paclitaxel	69
							Paclitaxel	181
KEYNOTE-522 (3)	Double-blind	III	TNBC	Early BC	cT1 cN1–2 or T2-4cN0-2	pCR, EFS	Pembrolizumab + carboplatin + paclitaxel	784
							Carboplatin + paclitaxel	390
NCI 10013 (19)	Open-label	II	TNBC	Early BC	T2-T4c, M0	pCR	Atezolizumab + carboplatin + paclitaxel	45
							Carboplatin + paclitaxel	22
NeoTRIP (20)	Open-label	III	TNBC	Early/LA BC	cT1cN1, cT2cN1, cT3cN0	EFS	Atezolizumab + carboplatin + paclitaxel	138
							Carboplatin + paclitaxel	142
APTneo (11)	Open-label	III	HER2+	Early/LA BC	cT1cN1, cT2cN1, cT3cN0, or stage III BC	EFS	Atezolizumab + carboplatin + paclitaxel	448
							Carboplatin + paclitaxel	223
ABCSG-52 (10)	Open-label	II	HER2+	Early BC	cT1c to CT4a-d, N0-3, M0	pCR	Atezolizumab + trastuzumab + pertuzumab	29
							Trastuzumab + pertuzumab	29
CamRelief (12)	Double-blind	III	TNBC	Early/LA BC	T2N0-1M0/T3N0M0 or T2N2-3M0/T3N1-3M0	pCR	Camrelizumab + nab-paclitaxel + carboplatin	222
							Nab-paclitaxel + carboplatin	219
CheckMate 7FL (13)	Double-blind	III	HR+/HER2-	Early BC	cN1-cN2 or cT3-cT4, cN0-cN2	pCR	Nivolumab + paclitaxel	263
							Paclitaxel	258
KEYNOTE-756 (17)	Double-blind	III	HR+/HER2-	Early BC	cT1-2(≥2 cm) cN1–2 or T3–4 cN0-2	pCR, EFS	Pembrolizumab +paclitaxel	635
							Paclitaxel	643

BC, breast cancer; EFS, event-free survival; LA, locally advanced; pCR, pathological complete response; TNBC, triple-negative breast cancer.

Overall, there was a low risk of bias among the included RCTs (**Supplementary Figure 1**); the main issue was a lack of blinding since ABCSG-52 (10), APTneo (11), I-SPY2 (18), NCI 10013 (19), and NeoTRIP (20) were open-labeled.

### Overall efficacy of ICI-based neoadjuvant therapy

**Figure 2:**
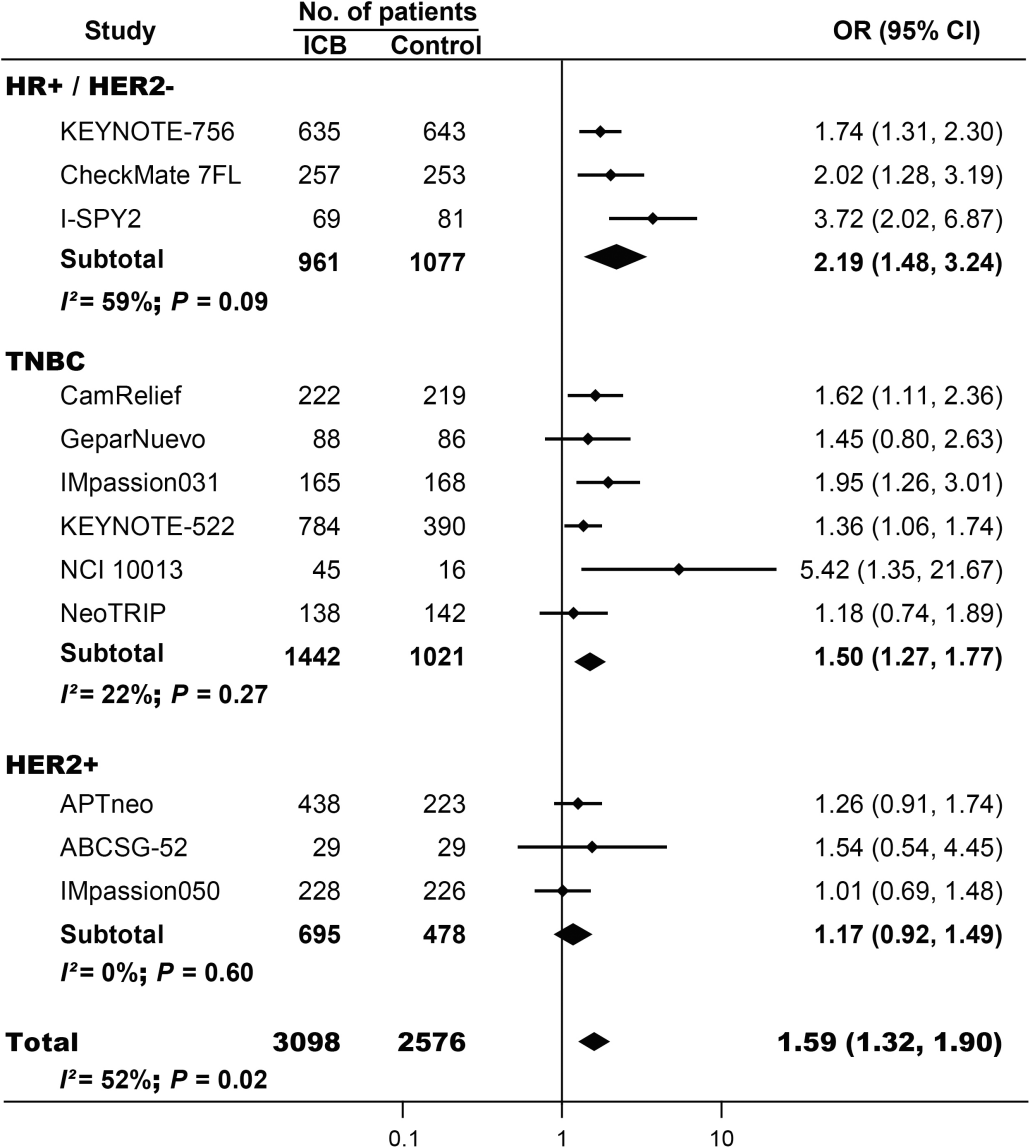
The pooled odds ratio of pathological complete response (pCR) in patients treated with ICI-based neoadjuvant regimens for breast cancer (BC). ICB, immune checkpoint blockade; OR, odds ratio; TNBC, triple negative breast cancer.

Totally, 1516 pCRs occurred among 3098 patients treated with neoadjuvant immunotherapy (incidence, 50.6%; 95% CI, 40.2%-61.0%), while 927 pCRs were identified in 2576 controls (incidence, 38.1%; 95% CI, 26.6%-50.4%). ICIs were associated with significantly increased pCRs (OR, 1.59; 95% CI, 1.32-1.90; *P* < 0.001; **Figure 2**). No significant asymmetry was identified by visual inspection of Begg’s funnel plot (**Supplementary Figure 2**). Notably, significant heterogeneity was observed among the included trials (*I^2^* = 52%, *P* = 0.02). Since the efficacy of neoadjuvant immunotherapy varies among different subtypes of BC, the FDA has currently only approved the application of neoadjuvant immunotherapy in TNBC. We further conducted subgroup analyses based on the subtypes of BC. ICI-based neoadjuvant therapy could significantly increase pCRs in women with HR+/HER2- BC (OR, 2.19; 95% CI, 1.48-3.24; *P* < 0.001) and TNBC (OR, 1.50; 95% CI, 1.27-1.77; *P* < 0.001), but not in individuals with HER2+ BC (OR, 1.17; 95% CI, 0.92-1.49; *P* = 0.23). Although there was still significant heterogeneity among trials conducted in HR+/HER2- BC, the benefits of immunotherapy were observed in every single trial. The heterogeneities just indicate the different extents of beneficiery.

The benefits of neoadjuvant immunotherapy were observed in both double-blind (OR, 1.52; 95% CI, 1.33-1.74; *P* < 0.001) and open-labeled trials (OR, 1.53; 95% CI, 1.21-1.94; *P* < 0.001; **Supplementary Figure 3**); phase III (OR, 1.46; 95% CI, 1.29-1.65; *P* < 0.001) and phase II RCTs (OR, 2.32; 95% CI, 1.59-3.40; *P* < 0.001; **Supplementary Figure 4**); early BC (OR, 1.60; 95% CI, 1.39-1.84; *P* < 0.001) and early/locally advanced BC (OR, 1.35; 95% CI, 1.09-1.68; *P* = 0.007; **Supplementary Figure 5**); trials enrolled over 400 patients (OR, 1.45; 95% CI, 1.27-1.65; *P* < 0.001) and trials with less than 400 women (OR, 1.83; 95% CI, 1.43-2.33; *P* < 0.001; **Supplementary Figure 6**), and ICIs targeting PD-1 (OR, 1.67; 95% CI, 1.44-1.94; *P* < 0.001) and ICIs targeting PD-L1 (OR, 1.34; 95% CI, 1.12-1.60; *P* = 0.002; **Supplementary Figure 7**). No significant asymmetry was observed by visual inspection of Begg’s funnel plot in all these subgroup analyses.

### Impact of nodal status or sTIL on neoadjuvant immunotherapy in breast cancer

**Figure 3:**
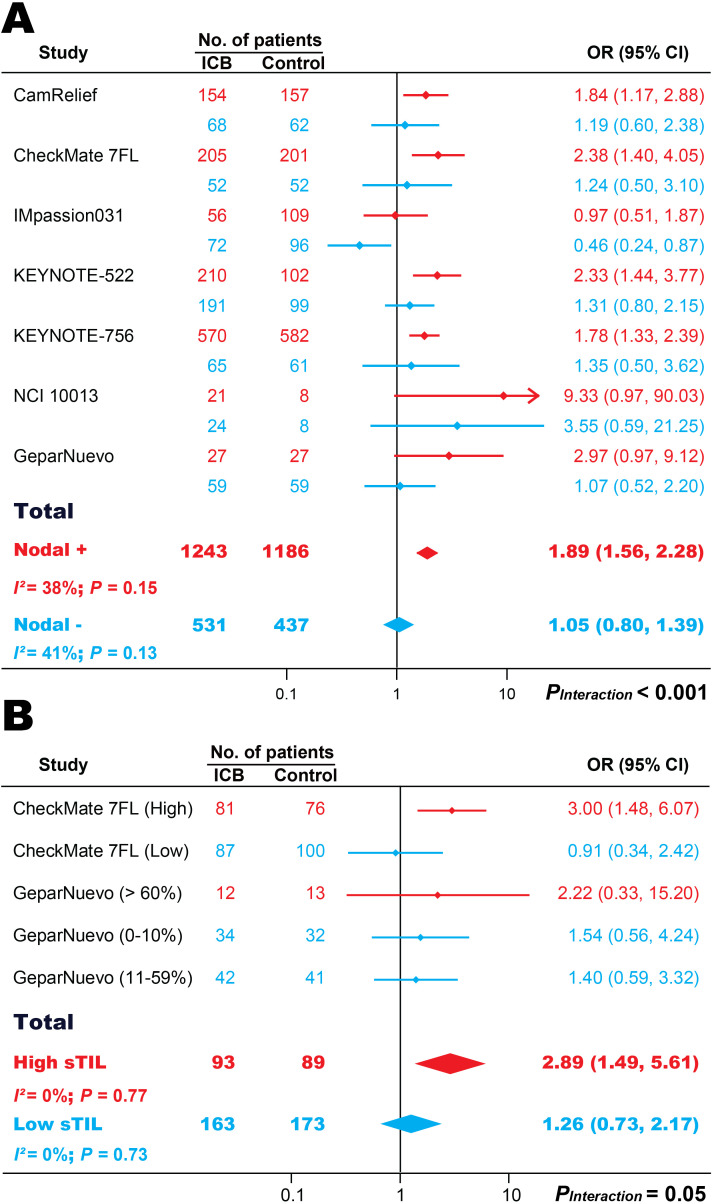
The association between nodal status **(A)** or stromal tumor-infiltrating lymphocyte **(B)** and pathological complete response in patients treated with ICI-based neoadjuvant regimens. ICB, immune checkpoint blockade; OR, odds ratio; sTIL, stromal tumor-infiltrating lymphocyte.

The nodal status was reported in 7 trials with 3397 patients (**Figure 3A**). ICI-based neoadjuvant therapy was associated with more pCRs in 2429 women with nodal-positive BC (OR, 1.89; 95% CI, 1.56-2.28; *P* < 0.001), but in 968 nodal-negative patients (OR, 1.05; 95% CI, 0.80-1.39; *P* = 0.71). There was a robust nodal status-treatment interaction (*P_Interaction_* < 0.001).

The enrichments of sTILs were examined in 2 studies with 518 women (**Figure 3B**). The addition of ICIs was associated with a higher pCR rate in 182 women with high-density sTILs (OR, 2.89; 95% CI, 1.49-5.61; *P* < 0.001), but in 336 patients with low-density sTILs (OR, 1.26; 95% CI, 0.73-2.17; *P* = 0.41). There was a statistically marginal sTIL-treatment interaction (*P_Interaction_* = 0.05).

No significant heterogeneities were observed in either of the two subgroup analyses (**Figure 3**).

### Association between neoadjuvant immunotherapy and other clinicopathological and molecular factors in breast cancer

The PD-L1 status was recorded in 10 trials with 4148 patients (**Supplementary Figure 8**). Neoadjuvant immunotherapy improved efficacy in both 2608 women with PD-L1-positive BC (OR, 1.73; 95% CI, 1.47-2.05; *P* < 0.001) and 1540 patients with PD-L1-negative tumors (OR, 1.41; 95% CI, 1.10-1.80; *P* = 0.01). The benefits were similar between these two subgroups (*P_interaction_* = 0.27).

The clinical stage was identified in 4 RCTs with 2547 women (**Supplementary Figure 9**). ICI-based neoadjuvant therapy was associated with more pCRs in 1618 patients with stage II BC (OR, 1.71; 95% CI, 1.36-2.14; *P* < 0.001) and 929 stage III subjects (OR, 2.00; 95% CI, 1.45-2.76; *P* < 0.001). The efficacy was independent of clinical stage (*P_interaction_* = 0.46).

The HR status was reported in 4 RCTs with 2972 women (**Supplementary Figure 10**). ICI-based neoadjuvant therapy increased the pCR rates in both 628 patients with HR-low tumors (OR, 2.45; 95% CI, 1.72-3.49; *P* < 0.001) and 2344 subjects with HR-high BC (OR, 1.51; 95% CI, 1.23-1.87; *P* < 0.001). Surprisingly, women with HR-low tumors benefited more from neoadjuvant immunotherapy (*P_interaction_* = 0.02).

Age information was found in 3 trials with 948 patients (**Supplementary Figure 11**). The addition of ICIs was associated with a higher pCR rate in both 221 women <40 years old (OR, 1.96; 95% CI, 1.14-3.37) and 727 patients over 40 years old (OR, 1.60; 95% CI, 1.19-2.15). Menopausal status was recorded in 2 RCTs with 1331 women (**Supplementary Figure 12**). Neoadjuvant immunotherapy improved pCRs in both 730 pre-menopausal patients (OR, 1.57; 95% CI, 1.09-2.28) and 601 post-menopausal patients (OR, 2.09; 95% CI, 1.38-3.17). The benefits of ICI-based neoadjuvant therapy were consistent regardless of age (*P_interaction_* = 0.52) and menopausal status (*P_interaction_* = 0.36).

Similar analyses were also conducted on 3 studies involving 1165 patients with race information (770 white women vs. 395 Asian women; **Supplementary Figure 13**), 4 RCTs enrolled 2652 patients with Eastern Cooperative Oncology Group (ECOG) performance status (2351 women with ECOG = 0 vs. 301 women with ECOG = 1; **Supplementary Figure 14**), and 3 trials recruited 2334 patients with known T stage (1560 women with T1–2 vs. 774 women with T3-4; **Supplementary Figure 15**). The efficacy of neoadjuvant immunotherapy was independent of race (*P_interaction_* = 0.76), ECOG status (*P_interaction_* = 0.54), and T stage (*P_interaction_* = 0.99).

## Discussion

This comprehensive review and meta-analysis based on 12 RCTs with 5674 women, for the first time to our knowledge, examined the association between the efficacy of neoadjuvant immunotherapy and routinely collected information on patients, diseases, and molecular features. Our results revealed that, compared with conventional neoadjuvant therapy, the addition of ICIs resulted in 1.59 times more pCRs in patients with breast cancer. Of note, the improvements were only observed in HR+/HER2- BC and TNBC, but not in HER2+ BC. Moreover, the interactions between neoadjuvant immunotherapy and nodal status or sTILs were statistically meaningful. Patients with nodal-positive or enriched sTIL tumors benefit significantly from ICI-based neoadjuvant therapy. In contrast, for women with nodal-negative or low-density sTIL BC, clinicians need to carefully balance efficacy, safety, and patient preferences to deliver individualized treatment. Additionally, this study did not provide sufficient evidence to recommend race, age, PD-L1 expression, clinical stage, HR status, menopausal status, ECOG performance status, or T stage as predictive biomarkers to guide patient selection for ICI-based neoadjuvant therapy in BC. These findings may aid in clinical trial design and interpretation, guide treatment decision-making, and thus promote personalized immunotherapy.

This meta-analysis is notable as previous studies did not reveal the association between various clinicopathological or molecular features and the efficacy of neoadjuvant immunotherapy in breast cancer (21, 22). Consistent with previous studies (21, 22), ICI-based neoadjuvant therapy was associated with favorable pCR in breast cancer. However, further analysis demonstrated that patients with HER2+ tumors failed to benefit from ICI-based neoadjuvant therapy. It should be noted that, in HER2+ BC with advanced or metastatic diseases, immunotherapy was also associated with poor clinical outcomes in several trials, including CCTG IND.229 (23), DS8201-A-U105 (24), JAVELIN Solid Tumor trial (25), KATE2 (26), and PANACEA (27). Currently, the interactions between anti-tumor immunity and HER2 expression are not completely understood (28). It was reported that HER2+ BC frequently demonstrated immunosuppressive characteristics with the tumor microenvironment, including the overexpression of immune checkpoint molecules and regulatory T cells (29). These immunosuppressive mechanisms could compromise antitumor immune responses, facilitating immune evasion and resistance to treatment. Accordingly, the modulation of the HER2 signaling pathway may affect immune regulation within the tumor microenvironment and strengthen anti-tumor immune responses.

Our analysis demonstrated evidence to recommend nodal status and sTIL level as predictive biomarkers in neoadjuvant immunotherapy. A substantial amount of research indicates that lymph nodes are vital in managing anti-tumor immunity, and the activation of immune responses in lymph nodes is crucial for the success of immunotherapy (30). In several animal models, pre-clinical experiments revealed that immunotherapy becomes less effective when LN dissection is performed (31, 32). Pharmacologically preventing lymphocyte egress from lymph nodes with the sphingosine-1-phosphate receptor agonist FTY720 negates the efficacy of multiple immunotherapies (30, 31). Additionally, investigations into the uninvolved sentinel lymph nodes of melanoma patients suggested an increase in cellular mediators that could enhance anti-tumor immune responses (33). Although the exact processes that lead to enhanced anti-tumor immunity in lymph nodes during immunotherapy are not fully understood, accumulating evidence suggests that certain subsets of CD8 T cells need to be mobilized from these lymph nodes to the tumor to initiate therapeutic responses (31, 32). It was reported that patients treated with neoadjuvant anti-PD-1 agents showed significant rises in tumor-specific T cell clones in the lymph nodes in operable lung cancer (34).

TILs, consisting of a diverse group of immune cells like CD3+ T-cells (CD8+ and CD4+), FOXP3+ T_reg_ cells, and CD20+ B cells, could offer useful insights into the patients’ baseline immune status (1). An increasing number of studies highlight the importance of certain TIL markers in evaluating immune responses and underscore their prognostic value in immunotherapy in breast cancer, melanoma, colorectal cancer, endometrial cancer, and non-small cell lung cancer (35). Higher baseline TIL density is associated with favorable outcomes such as overall survival (OS) and objective response rate in patients treated with ICIs (35, 36). Our study also revealed the density of sTIL as a predictive marker in neoadjuvant immunotherapy for breast cancer. Notably, the sTIL-treatment interaction was only statistically marginal. This is partly because the number of subjects enrolled in the meta-analysis was limited, and the methods of evaluating sTIL were different across medical centers. Additionally, it was reported that the reaction to ICI is also shaped by how TILS are spatially dispersed (35). Interestingly, even though it is not conclusive as a prognostic biomarker, growing evidence suggests that TIL subsets (e.g., CD8+, CD4+, T_regs_, and T_memory_), exhaustion markers such as PD-1, LAG-3, and TIM-3, and activation markers like granzyme B were related to clinical outcomes in various tumors (37).

Our findings showed that ICI-based neoadjuvant treatments enhanced pCRs in breast cancer regardless of PD-L1 expression status. Indeed, comparable outcomes had been observed in other cancers (38). It is well-known that PD-L1 expression status by itself is insufficient to determine which patients with advanced solid tumors should receive ICIs (39, 40). It seems more vital to determine the activity of the PD-1/PD-L1 pathway than to concentrate only on PD-L1 expression. According to Cancer Immunogram (41), the success of immunotherapy depends on a range of unrelated factors, including how ‘foreign’ the cancer appears, the status of the immune system, the existence of other inhibitory mechanisms, the activity of the immune system, the activity of T cell infiltrates within the tumor, and the tumor cells’ sensitivity to immune cells.

This study has some limitations. First, all the results here were derived from subgroup analysis. It was well-established that subgroup analysis, created retrospectively from RCT data, can introduce bias by its nature and is not dependable until it is confirmed by further studies (42). However, the reasoning for conducting *post hoc* subgroup analysis is significant: when chosen correctly and methodologically sound, it can serve as a valid hypothesis testing framework when grounded in prior empirical evidence and current scientific theories. Moreover, although the imbalance of different subgroups could introduce bias, fortunately, most subgroup analyses conducted in this study provide balance between intervention arms. Second, some subgroup analyses are based on a limited number of trials, involve heterogeneous definitions and assessment methods, and in some cases demonstrate only marginally significant interaction tests. Accordingly, those findings should be interpreted cautiously, and further randomized trials are essential to confirm their validity. Third, while conducting a meta-analysis of RCTs with overall survival as the primary endpoint would be ideal, it necessitates a considerable amount of time to gather enough survival information, even though breast cancer is an aggressive disease. Therefore, our study mainly focuses on pCR instead of survival. It should be noted that, although pCR is a commonly used endpoint in neoadjuvant trials, its validity as a surrogate for long-term outcomes is context-dependent, particularly in hormone receptor-positive disease (43) and in the setting of immunotherapy (44). Hence, trials with overall survival information were needed to validate our conclusions.

In conclusion, HER2 expression, nodal status, and sTIL levels were identified as potential predictive biomarkers to guide patient selection for neoadjuvant immunotherapy. For women with HER2-positive, low-level sTIL, or nodal-negative tumors, clinicians need to carefully balance efficacy, safety, and patient preferences to deliver individualized treatment. Of note, these findings should be interpreted cautiously due to the nature of *post hoc* subgroup analysis. Further research should keep evaluating the advantages of neoadjuvant immunotherapy by subgroup as overall survival data becomes more complete.

## Data Availability

The original contributions presented in the study are included in the article/**Supplementary Material**. Further inquiries can be directed to the corresponding authors.

## Supplementary material

Supplementary Figure 1 Risk of bias of eligible randomized trials assessed by the Cochrane risk of bias tool.

Supplementary Figure 2 The pooled OR of pCR **(A)** and publication bias **(B)** in double-blind and open-label trials.

Supplementary Figure 3 Begg’s funnel plot for the publication bias test. Each circle represents a separate trial for the indicated association. Vertical line, mean effect size.

Supplementary Figure 4 The pooled OR of pCR **(A)** and publication bias **(B)** in phase III and phase II trials.

Supplementary Figure 5 The pooled OR of pCR **(A)** and publication bias **(B)** in patients with early breast cancer and patients with early or locally advanced breast cancer.

Supplementary Figure 6 The pooled OR of pCR **(A)** and publication bias **(B)** in trials with over 400 patients and trials with fewer than 400 patients.

Supplementary Figure 7 The pooled OR of pCR **(A)** and publication bias **(B)** in trials with inhibitors targeting PD-1 and trials with inhibitors targeting PD-L1.

Supplementary Figure 8 The association between PD-L1 status and pCR in patients treated with ICI-based neoadjuvant regimens. **(A)** The pooled OR of pCR; **(B)** The publication bias.

Supplementary Figure 9 The association between clinical stage and pCR in patients treated with ICI -based neoadjuvant regimens. **(A)** The pooled OR of pCR; **(B)** The publication bias.

Supplementary Figure 10 The association between hormone receptor (HR) status and pCR in patients treated with ICI -based neoadjuvant regimens. **(A)** The pooled OR of pCR; **(B)** The publication bias.

Supplementary Figure 11 The association between age and pCR in patients treated with ICI -based neoadjuvant regimens. **(A)** The pooled OR of pCR; **(B)** The publication bias.

Supplementary Figure 12 The association between menopausal status and pCR in patients treated with ICI -based neoadjuvant regimens. **(A)** The pooled OR of pCR; **(B)** The publication bias.

Supplementary Figure 13 The association between race and pCR in patients treated with ICI -based neoadjuvant regimens. **(A)** The pooled OR of pCR; **(B)** The publication bias.

Supplementary Figure 14 The association between Eastern Cooperative Oncology Group (ECOG) performance status and pCR in patients treated with ICI -based neoadjuvant regimens. **(A)** The pooled OR of pCR; **(B)** The publication bias.

Supplementary Figure 15 The association between T stage and pCR in patients treated with ICI-based neoadjuvant regimens. **(A)** The pooled OR of pCR; **(B)** The publication bias.
