# Supplementary figures and images for: Clinicopathological and molecular characteristics associated with pathological complete response in neoadjuvant immunotherapy for breast cancer
Source: Front Immunol. 2026 Mar 27;17:1771228. doi: 10.3389/fimmu.2026.1771228 (PMC13066237; doi:10.3389/fimmu.2026.1771228)

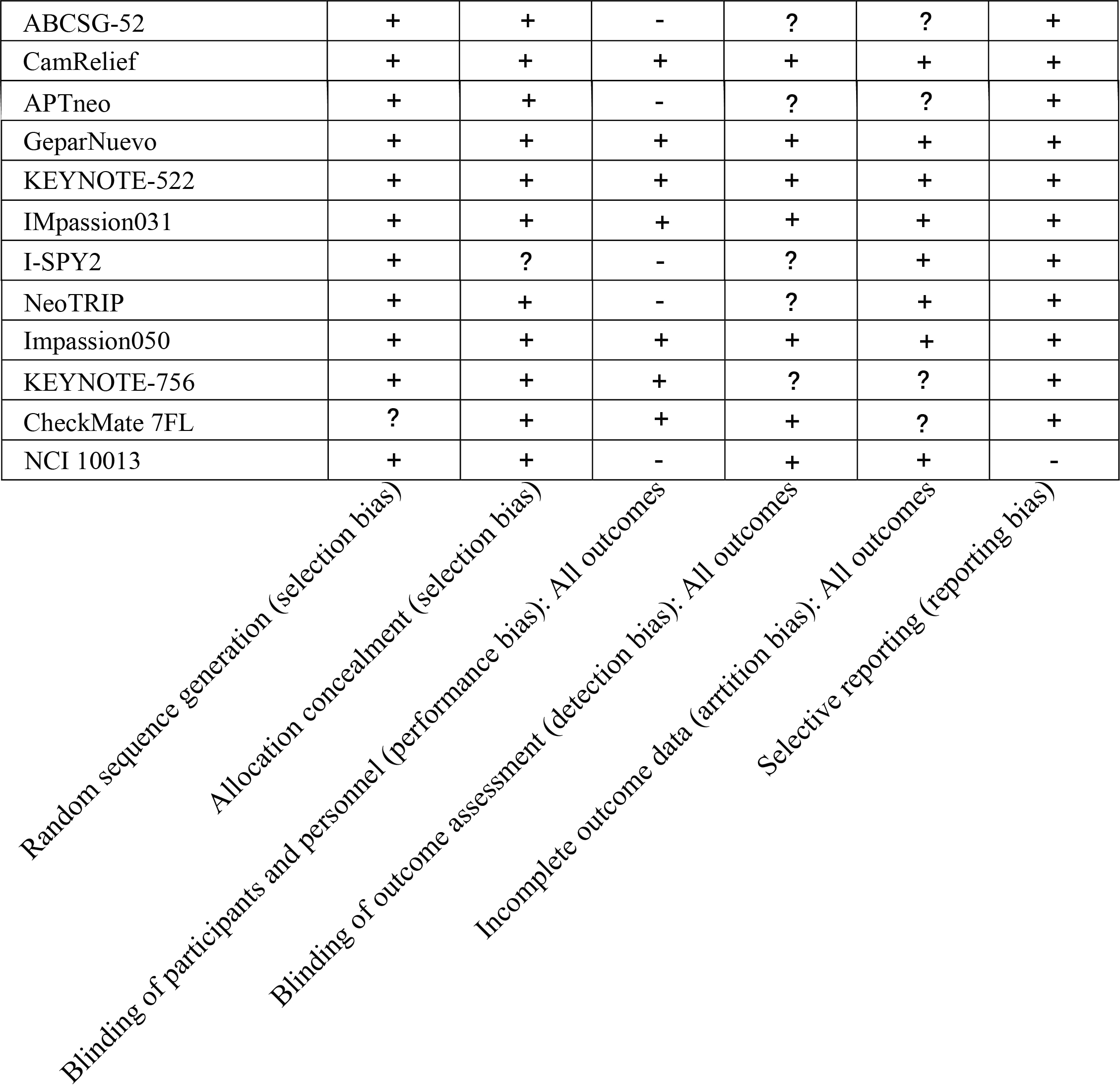

Supplement: Supplementary Figure 1 — Risk of bias of eligible randomized trials assessed by the Cochrane risk of bias tool. [file Image1.jpeg]

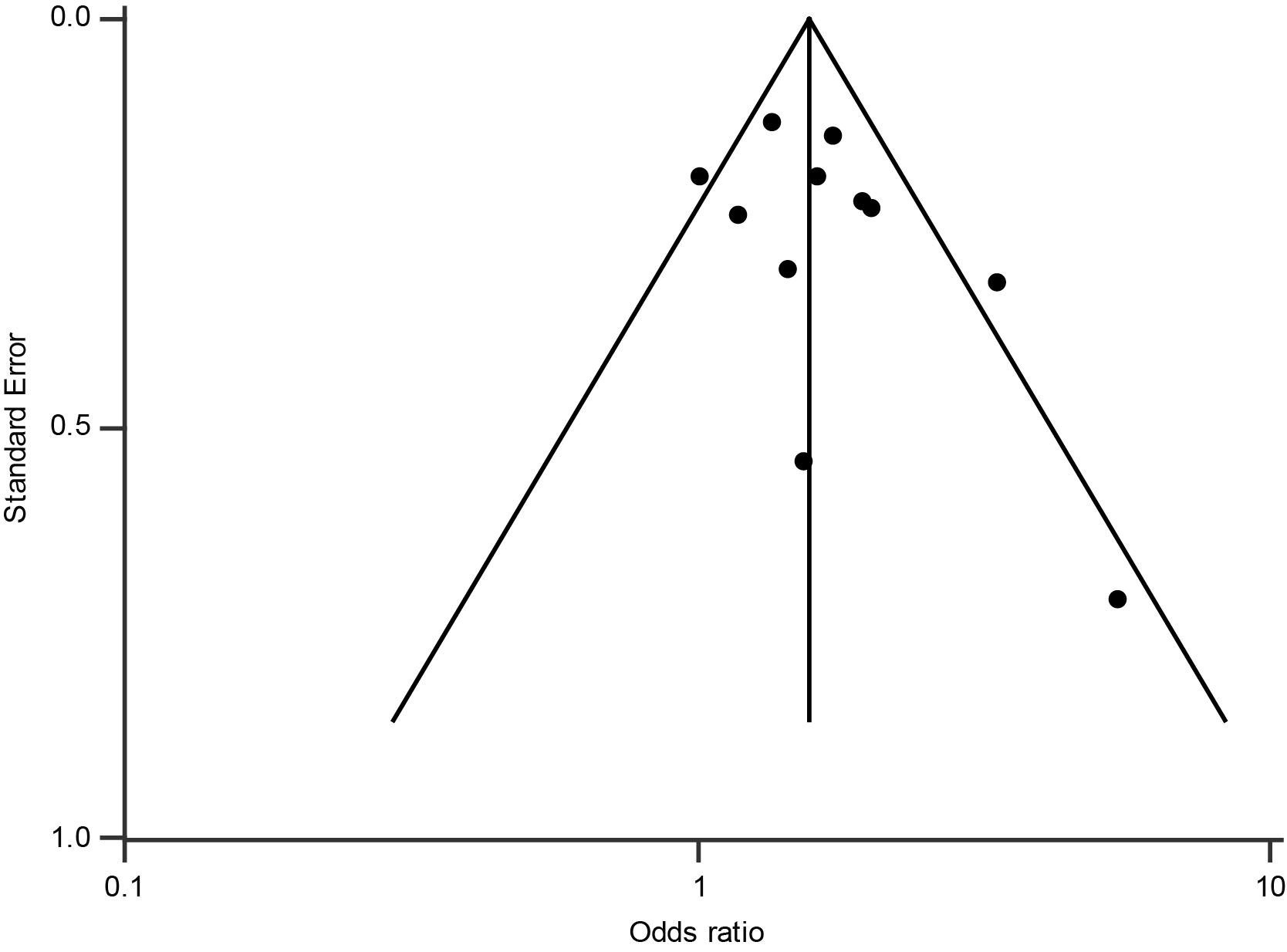

Supplement: Supplementary Figure 2 — The pooled OR of pCR (A) and publication bias (B) in double-blind and open-label trials. [file Image2.jpeg]

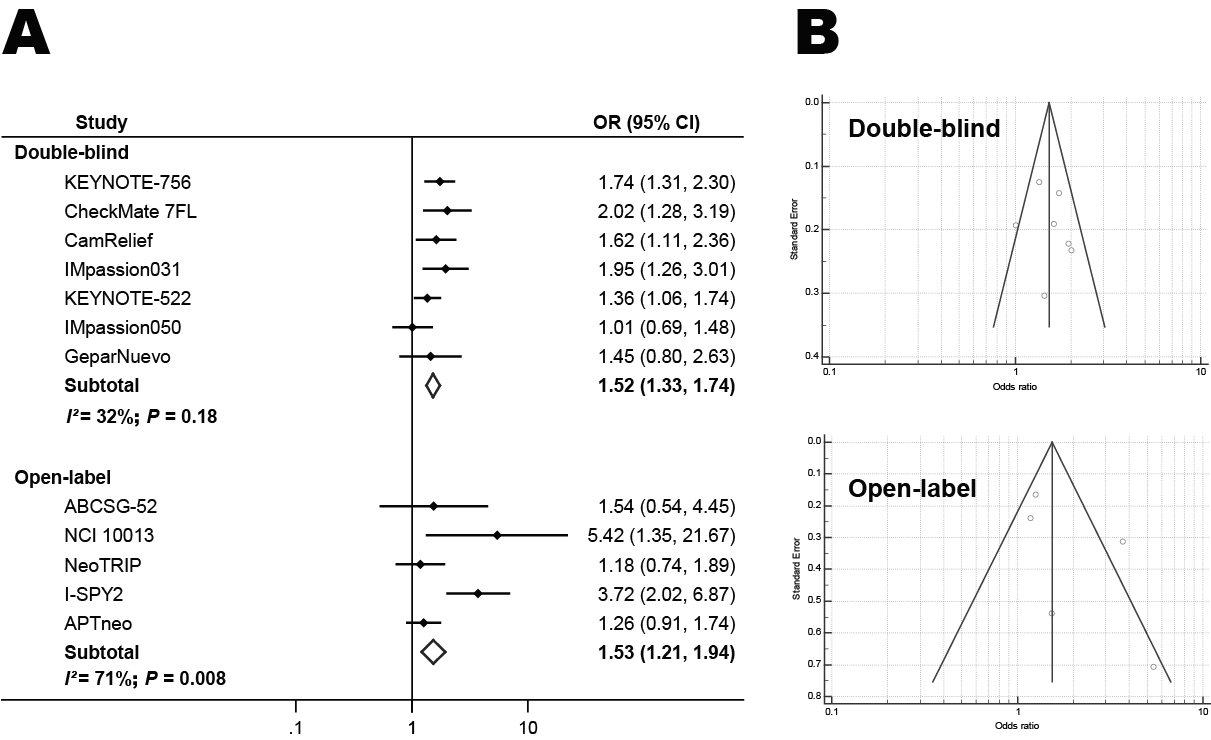

Supplement: Supplementary Figure 3 — Begg’s funnel plot for the publication bias test. Each circle represents a separate trial for the indicated association. Vertical line, mean effect size. [file Image3.jpeg]

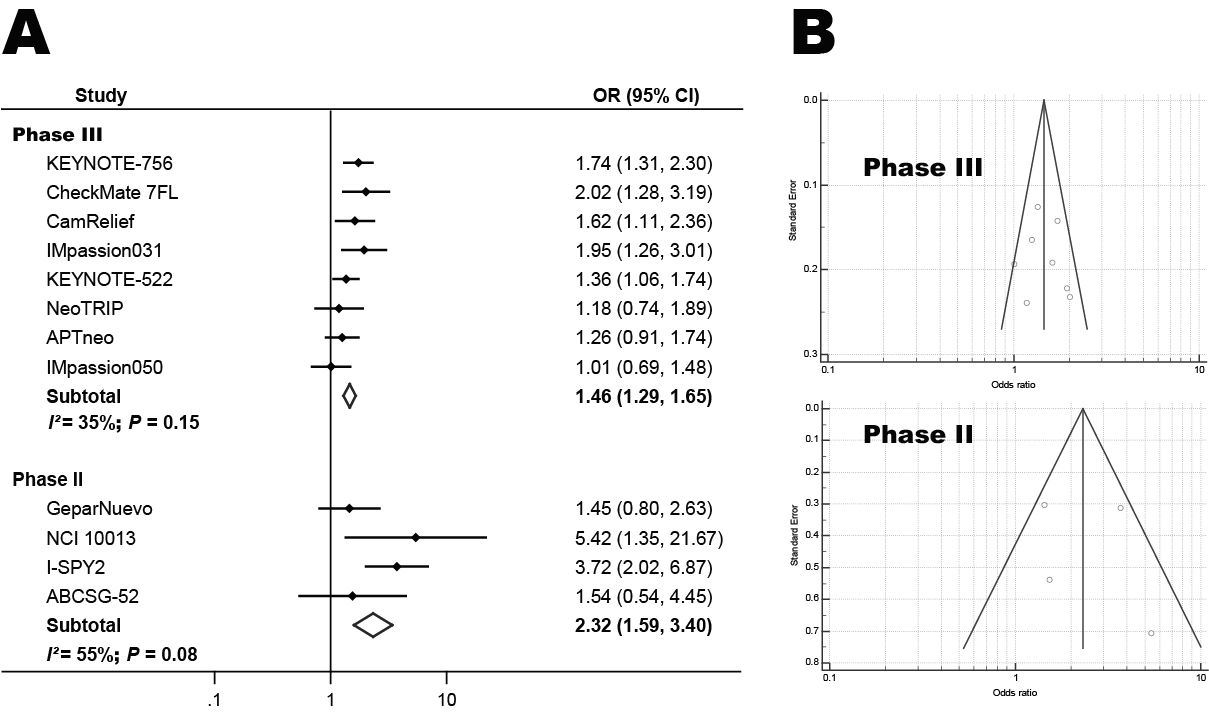

Supplement: Supplementary Figure 4 — The pooled OR of pCR (A) and publication bias (B) in phase III and phase II trials. [file Image4.jpeg]

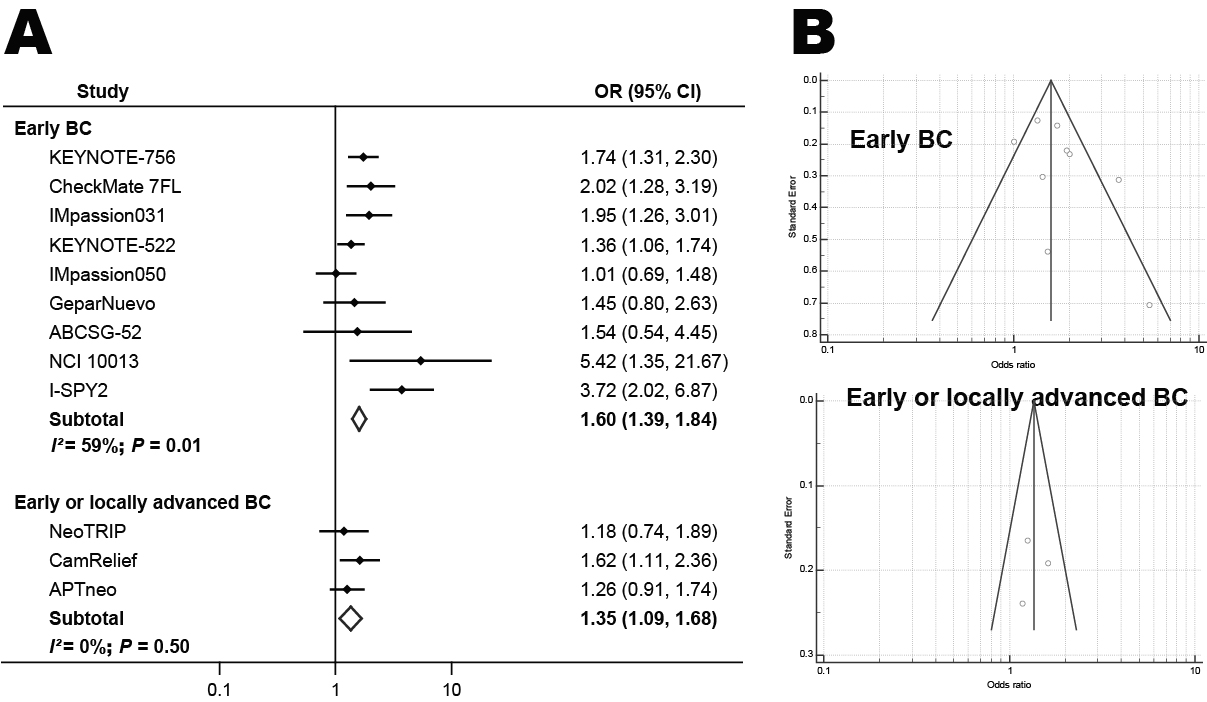

Supplement: Supplementary Figure 5 — The pooled OR of pCR (A) and publication bias (B) in patients with early breast cancer and patients with early or locally advanced breast cancer. [file Image5.jpeg]

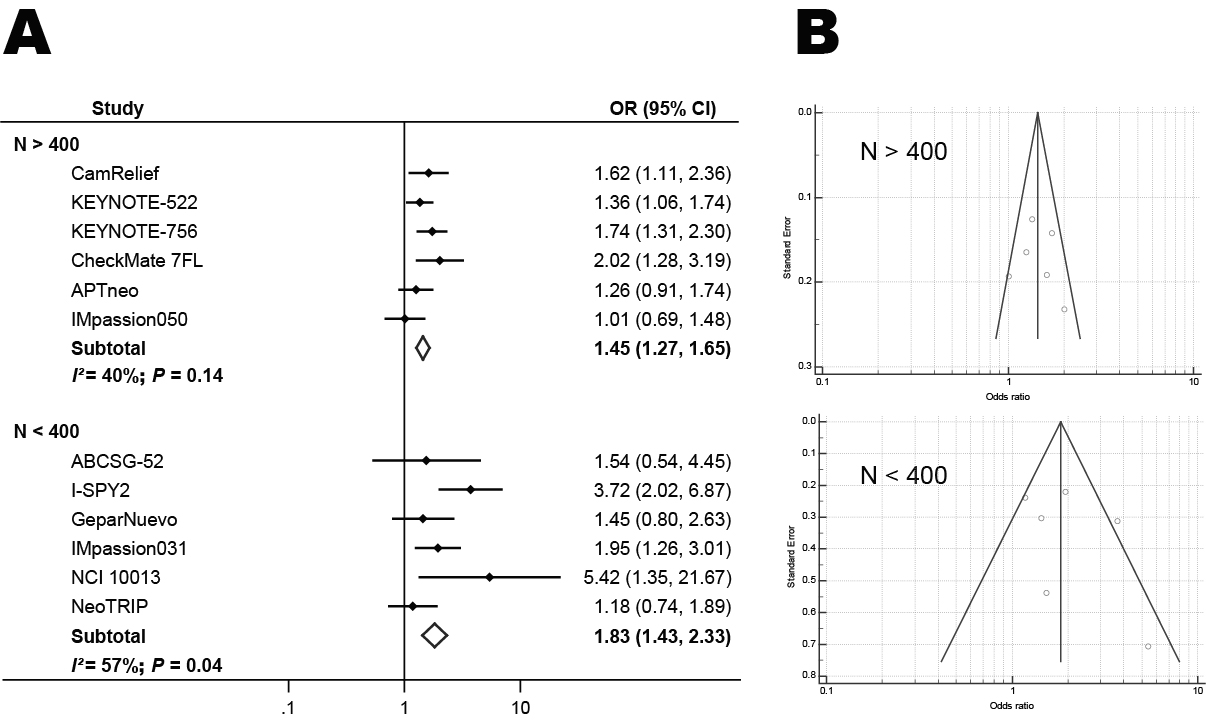

Supplement: Supplementary Figure 6 — The pooled OR of pCR (A) and publication bias (B) in trials with over 400 patients and trials with fewer than 400 patients. [file Image6.jpeg]

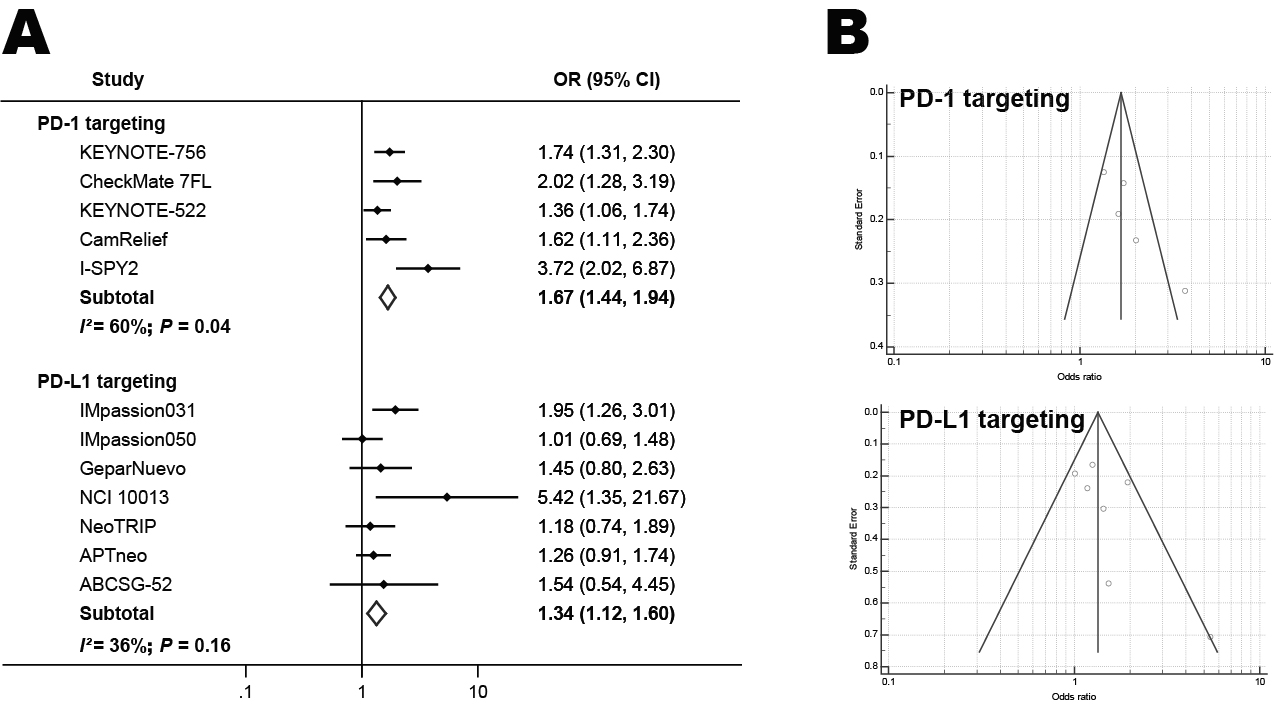

Supplement: Supplementary Figure 7 — The pooled OR of pCR (A) and publication bias (B) in trials with inhibitors targeting PD-1 and trials with inhibitors targeting PD-L1. [file Image7.jpeg]

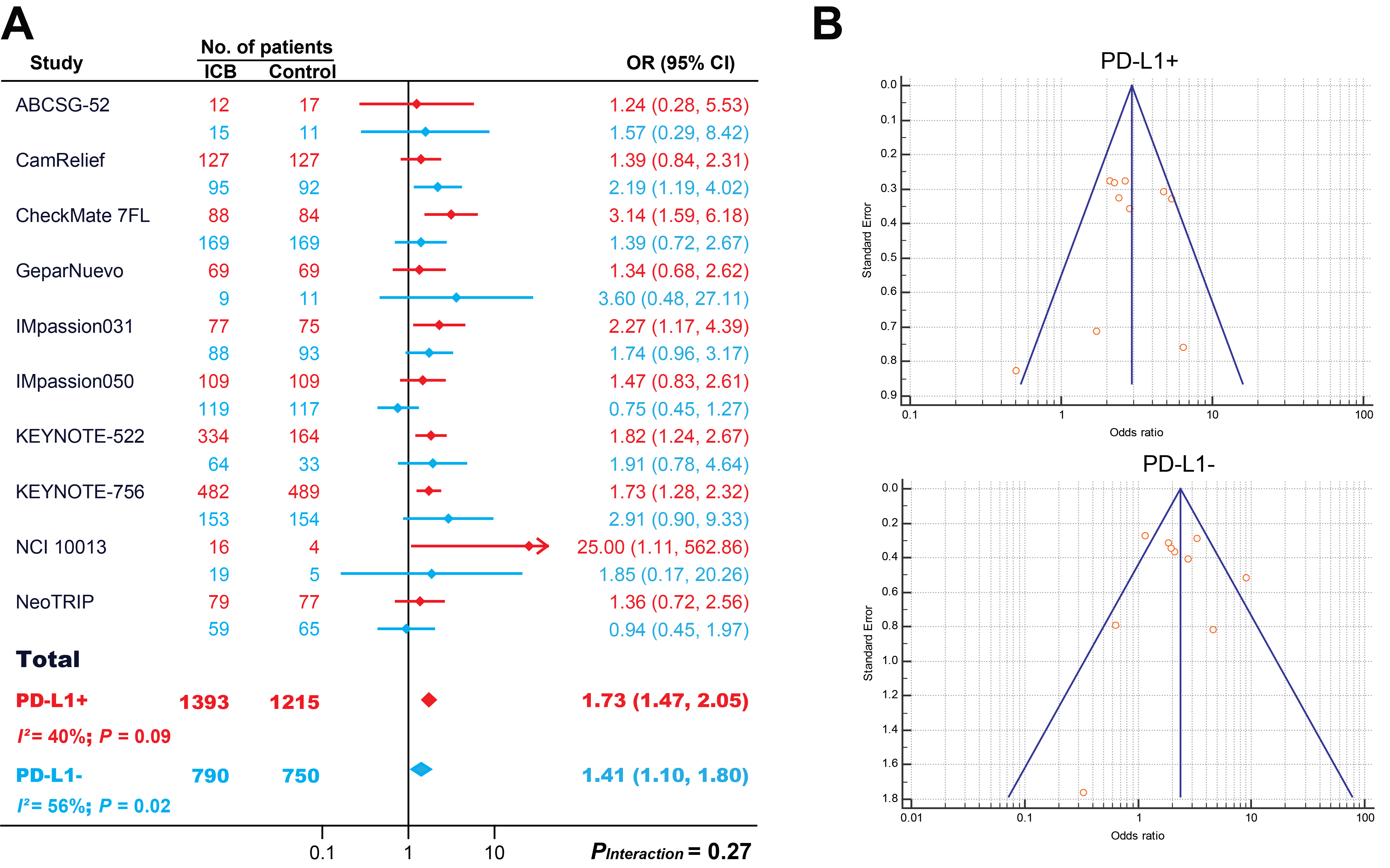

Supplement: Supplementary Figure 8 — The association between PD-L1 status and pCR in patients treated with ICI-based neoadjuvant regimens. (A) The pooled OR of pCR; (B) The publication bias. [file Image8.jpeg]

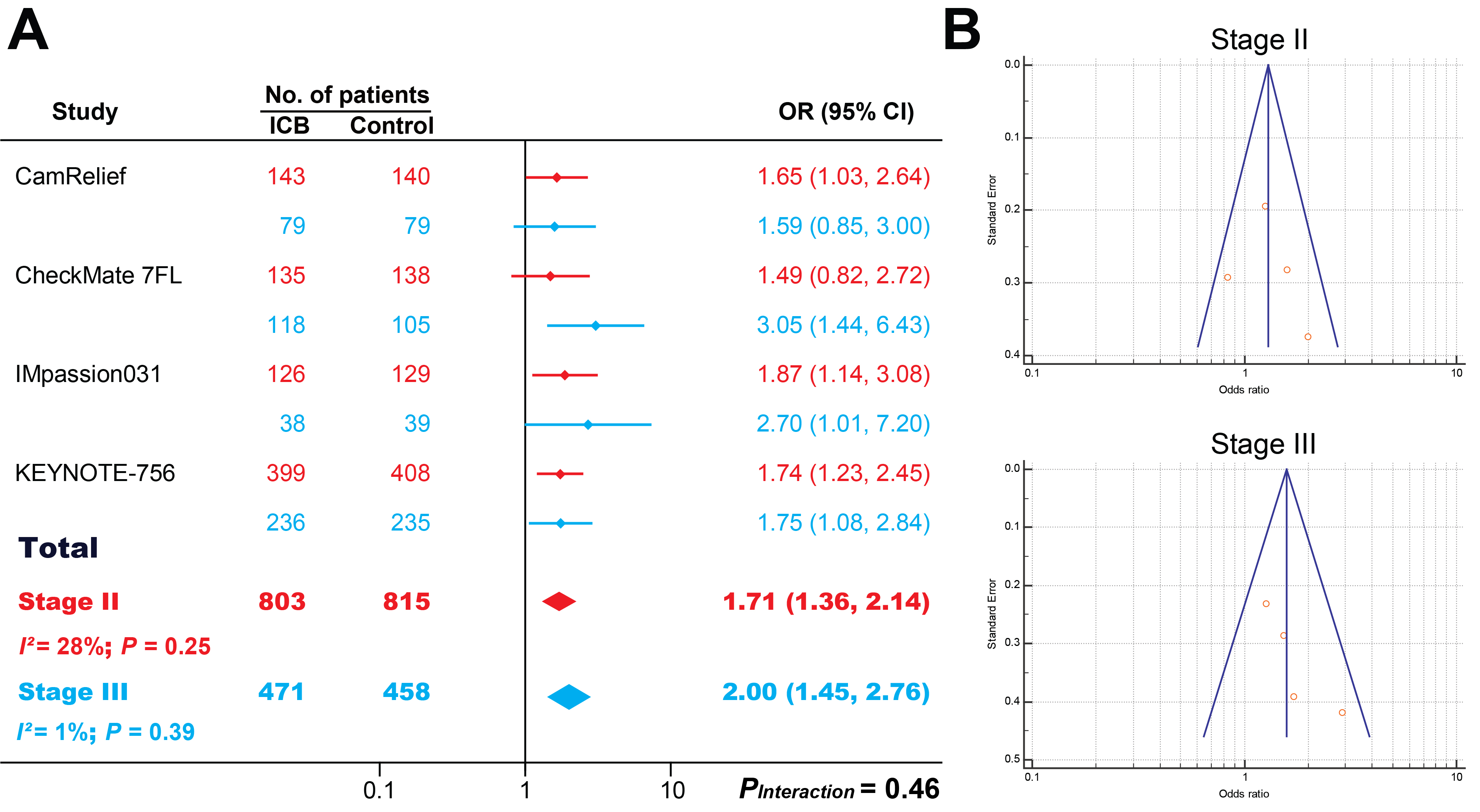

Supplement: Supplementary Figure 9 — The association between clinical stage and pCR in patients treated with ICI -based neoadjuvant regimens. (A) The pooled OR of pCR; (B) The publication bias. [file Image9.jpeg]

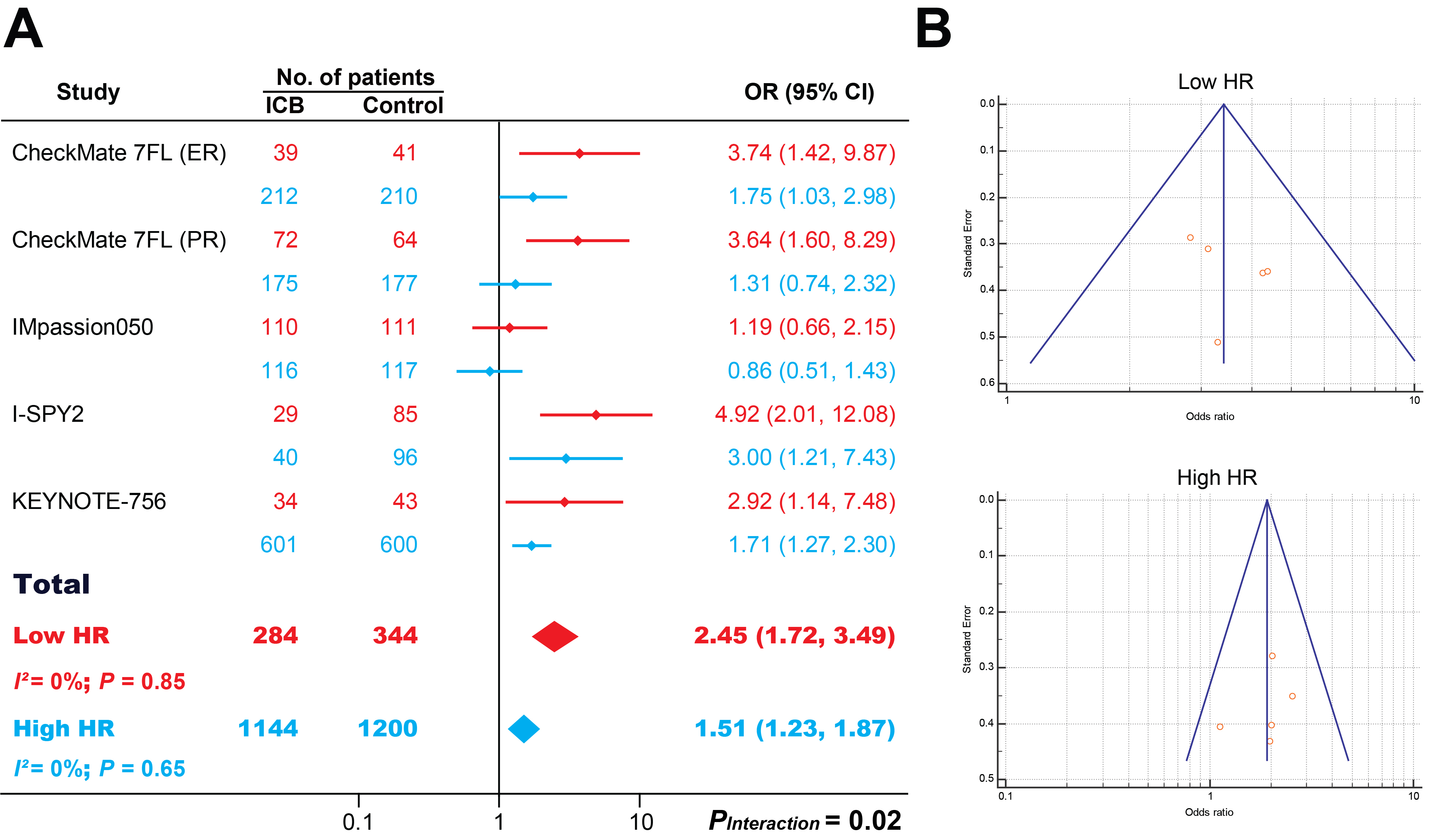

Supplement: Supplementary Figure 10 — The association between hormone receptor (HR) status and pCR in patients treated with ICI -based neoadjuvant regimens. (A) The pooled OR of pCR; (B) The publication bias. [file Image10.jpeg]

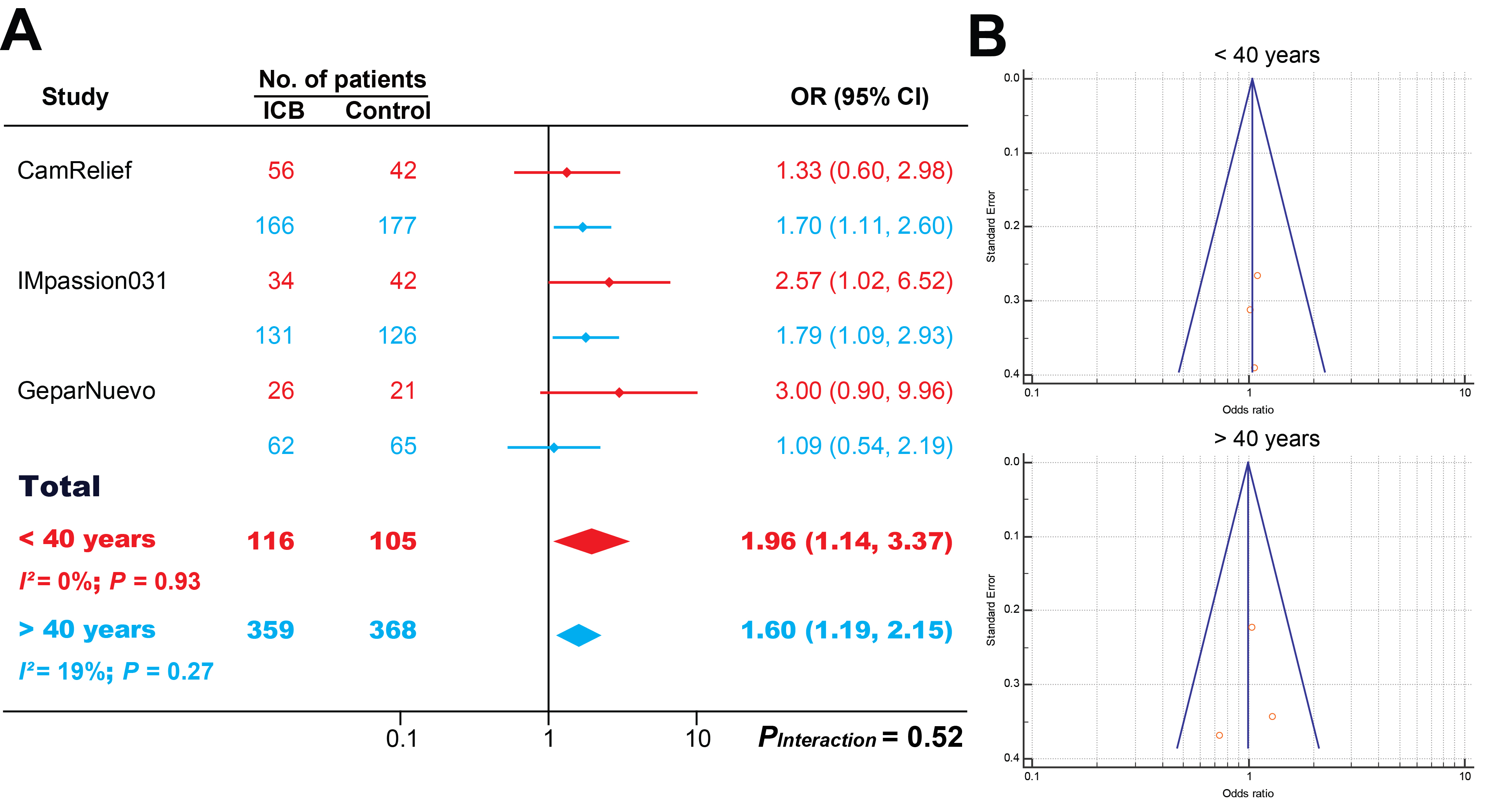

Supplement: Supplementary Figure 11 — The association between age and pCR in patients treated with ICI -based neoadjuvant regimens. (A) The pooled OR of pCR; (B) The publication bias. [file Image11.jpeg]

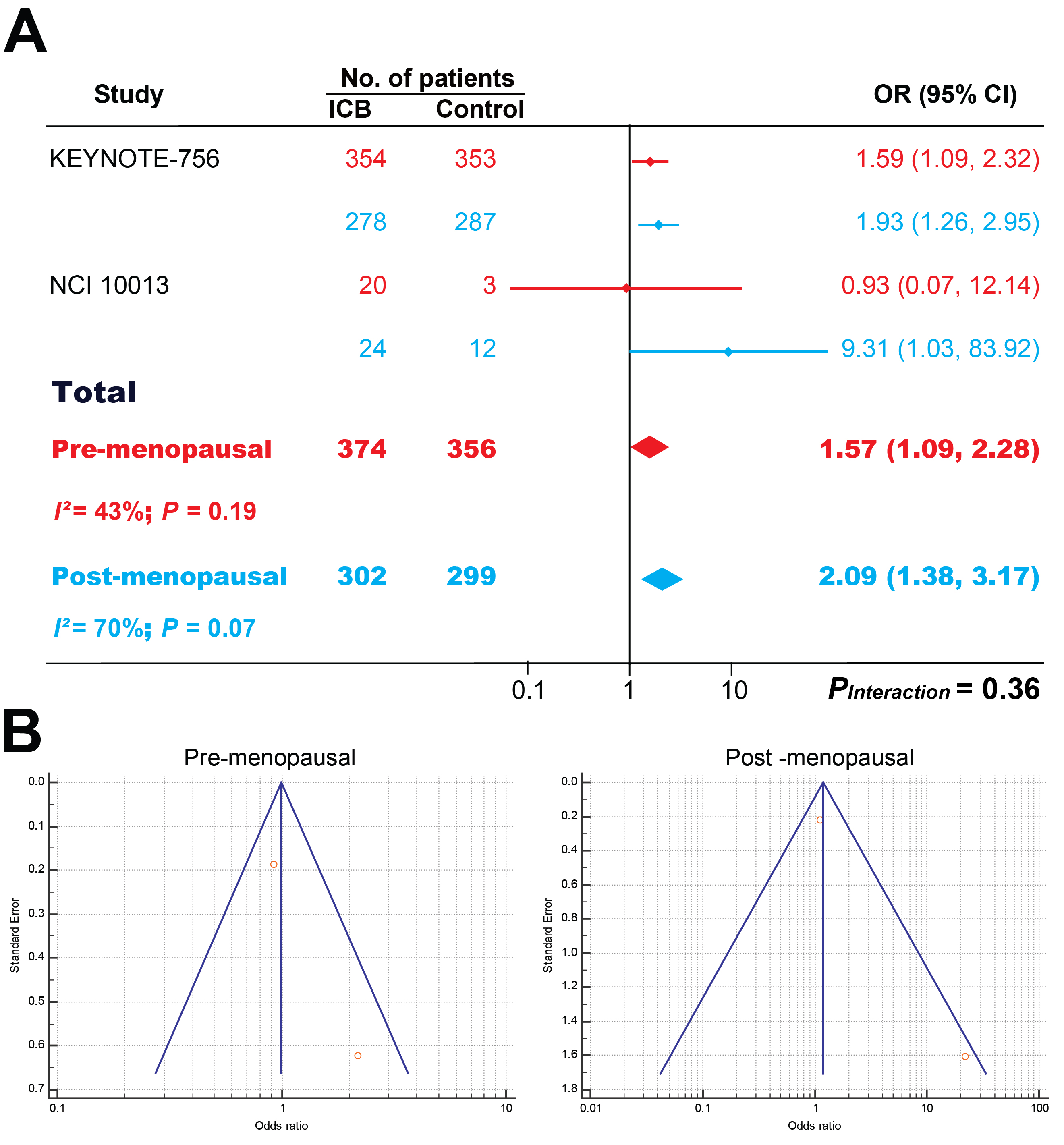

Supplement: Supplementary Figure 12 — The association between menopausal status and pCR in patients treated with ICI -based neoadjuvant regimens. (A) The pooled OR of pCR; (B) The publication bias. [file Image12.jpeg]

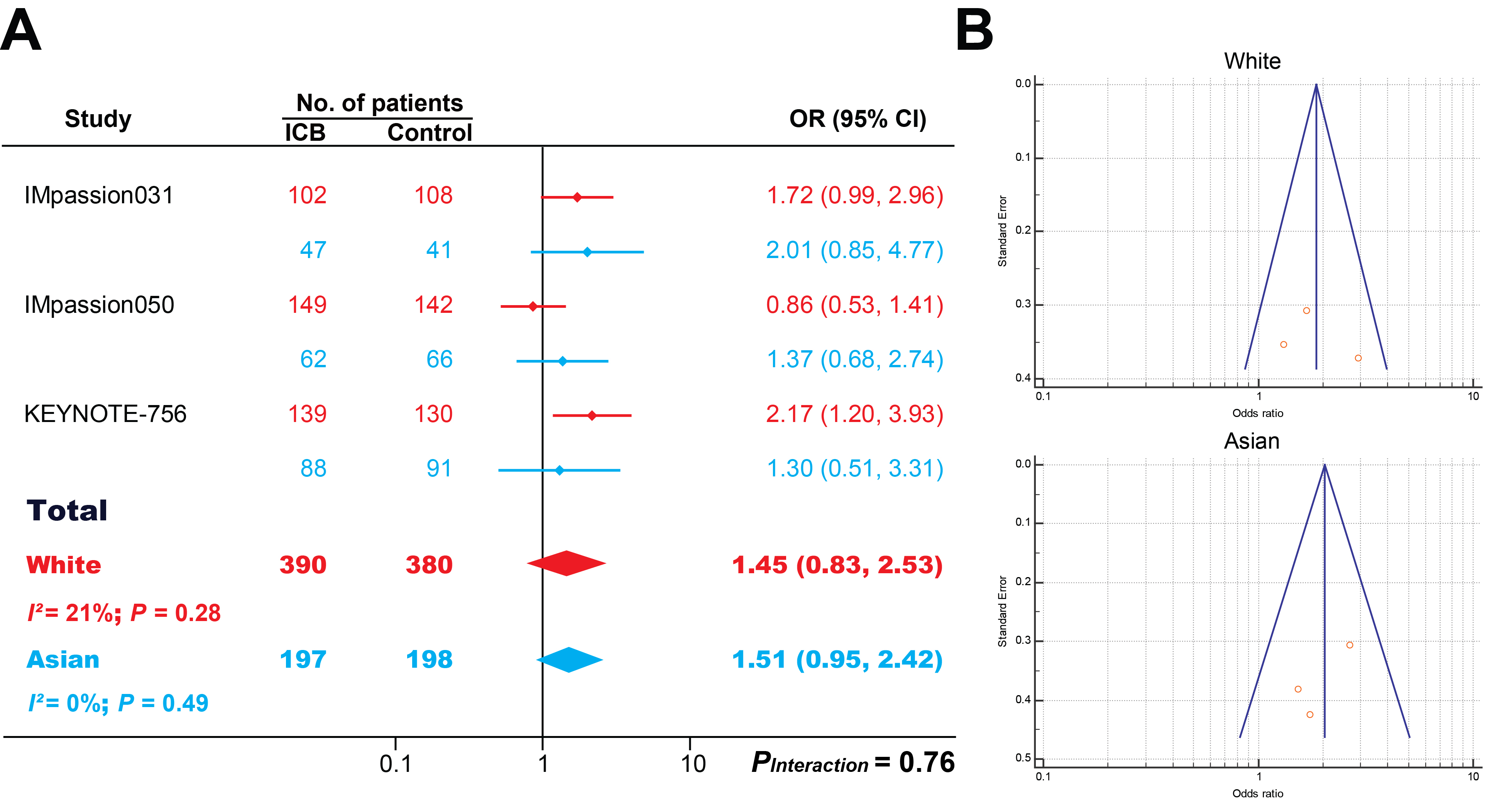

Supplement: Supplementary Figure 13 — The association between race and pCR in patients treated with ICI -based neoadjuvant regimens. (A) The pooled OR of pCR; (B) The publication bias. [file Image13.jpeg]

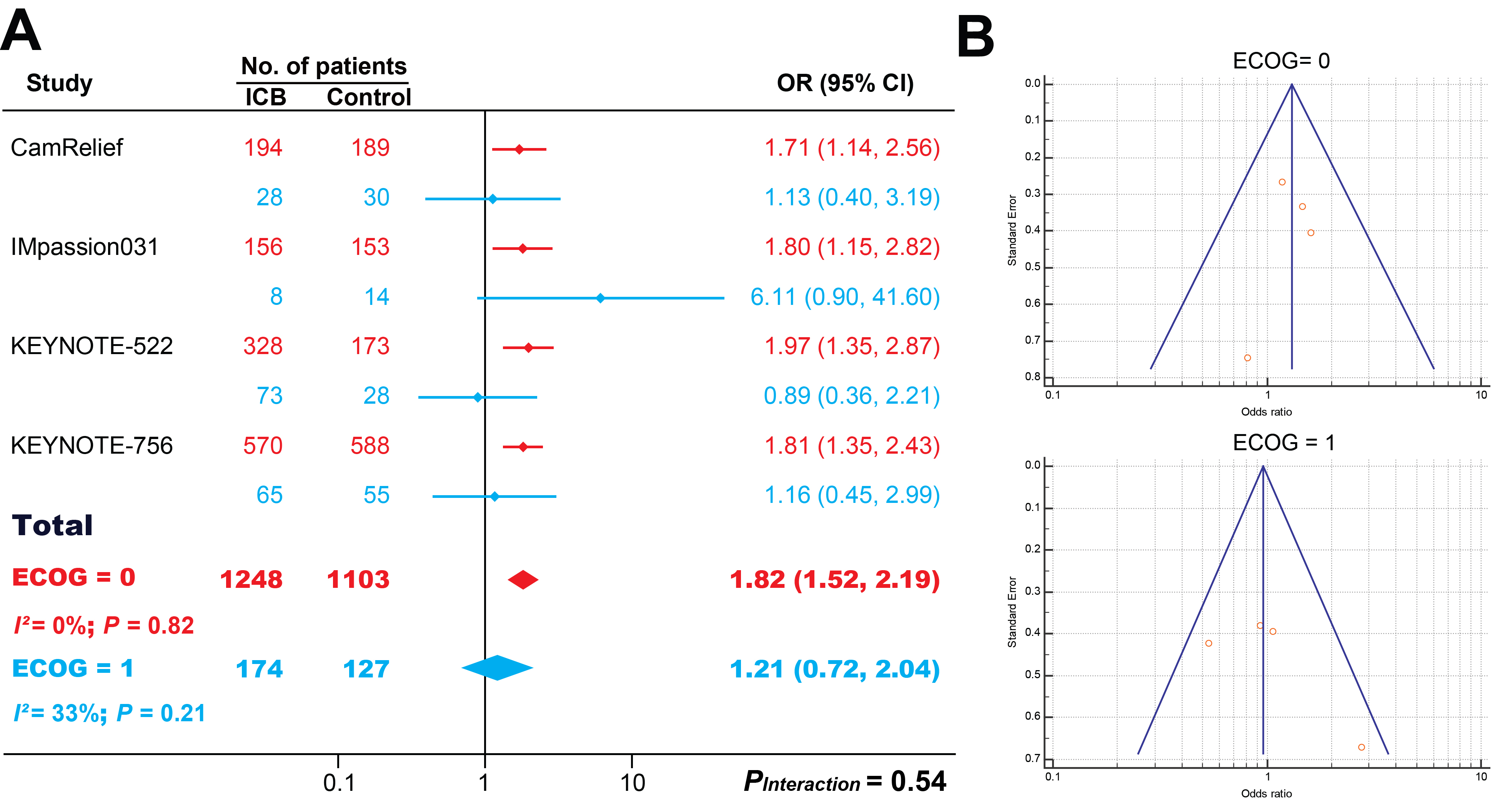

Supplement: Supplementary Figure 14 — The association between Eastern Cooperative Oncology Group (ECOG) performance status and pCR in patients treated with ICI -based neoadjuvant regimens. (A) The pooled OR of pCR; (B) The publication bias. [file Image14.jpeg]

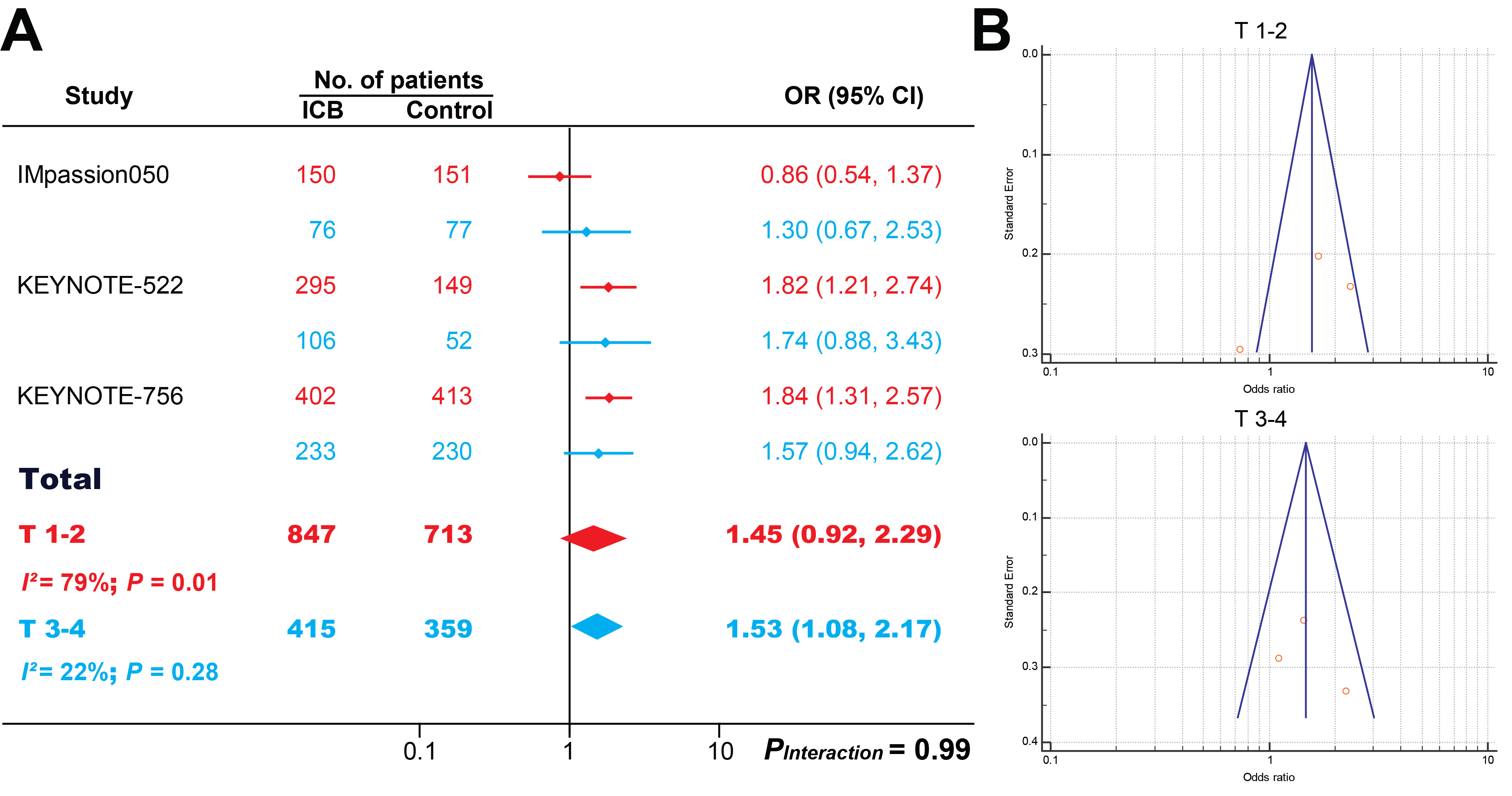

Supplement: Supplementary Figure 15 — The association between T stage and pCR in patients treated with ICI-based neoadjuvant regimens. (A) The pooled OR of pCR; (B) The publication bias. [file Image15.jpeg]
